# Comprehensive CAD/CAM Prosthetic Rehabilitation Management in a Young Patient with Agenesis: A Case Report

**DOI:** 10.3390/healthcare10020382

**Published:** 2022-02-17

**Authors:** Noémie Drancourt, Emmanuel Nicolas, Jean-Luc Veyrune, Marion Bessadet

**Affiliations:** 1 Centre de Recherche en Odontologie Clinique (CROC), Université Clermont Auvergne, 63000 Clermont-Ferrand, France; emmanuel.nicolas@uca.fr (E.N.); j-luc.veyrune@uca.fr (J.-L.V.); marion.bessadet@uca.fr (M.B.); 2 Service d’Odontologie, CHU Clermont-Ferrand, 63003 Clermont-Ferrand, France

**Keywords:** agenesia, CAD/CAM technology, full-mouth rehabilitation, special needs patients, oral health management

## Abstract

With the advancement in ceramic restorations bonded to the tooth structure, the treatment has become a practicable and conservative option to restore teeth with shape alteration owing to the high demand for aesthetics, with minimum tooth preparation. This article describes the case of a 25-year-old man who was dissatisfied with his smile. After an assessment of the clinical situation, a decision was made to place a full-mouth prosthodontic rehabilitation (monolithic lithium disilicate glass-ceramic, IPS E-max CAD) with minimal tooth preparation, which figures as a conservative full-coverage approach. The restoration of an aesthetic smile resulted in the patient’s satisfaction. Bonding of all monolithic veenerlay and crowns onto minimally prepared teeth figures as conservative treatment in cases of hypodontia.

## 1. Introduction

Root resorption associated with orthodontic treatment is an unintended iatrogenic side effect of multifactorial etiology that occurs in almost all orthodontic treatments. The mechanisms of root resorption are similar to those responsible for bone resorption, i.e., inflammatory pressure reactions. According to Bassigny, apical root resorption occurs in 50 to 60% of cases after orthodontic treatment. Root resorption is irreversible and difficult to avoid [1]. The presence of dental agenesis also leads to a moderate risk of resorption [2]. In the case of significant resorption, it is recommended to stop the current orthodontic treatment to review the treatment objectives and to favor a prosthetic alternative rather than closing spaces [3]. 

Computer-aided design (CAD)/computer-aided manufacturing (CAM) technology is one of the fastest-evolving aspects in modern restorative dentistry. The prosthetic management of these patients can be carried out by CAD/CAM technology, providing a minimally invasive approach. An increasing number of chairside systems are now available [4]. Intraoral scanners have become significantly better, faster, and smaller, with more intuitive design software surfaces. This virtual environment with on-screen designing and computer-assisted production with rapid prototyping, such as milling or the growing option of three-dimensional printing possibilities, allows for the fabrication of various restorations without any physical models.

The materials used in CAD/CAM technology combined with the appropriate bonding technique offer good long-term resistance and allow a satisfactory aesthetic result. In addition, the minor dental preparation required for this type of procedure is fully integrated into a conservative approach [5]. However, in order to optimize the long-term aesthetic and functional outcome of the treatment, it seems important to clearly define the treatment objectives as nowadays different ceramics can be used for the same indication [6].

The objective of this case report is to present a full-mouth prosthodontic therapeutic by CAD/CAM technology as an alternative to orthodontics in the case of multiple agenesis in a young patient.

## 2. Case Report

### 2.1. Case History

A 25-year-old man visited the prosthodontics department of the Odontology unit of the Clermont-Ferrand University Hospital following an aesthetic problem. He had no particular medical history. He complained about the shape of his teeth, the absence of some of them and his unaesthetic smile that he wanted to improve. The patient had been followed for 3 years by his orthodontist and treated with multi-attachments and bimaxillary retention trays during this period in order to close the spaces created by the agenesis. His oral quality of life was evaluated using the GOHAI (General Oral Health Assessment Index) questionnaire [7]. He obtained a score of 38/60, which corresponded to a poor oral quality of life. His masticatory performance was evaluated before and after prosthetic rehabilitation by the two-color chewing-gum test [8,9]. 

### 2.2. Diagnostic Assessment

**Figure 1:**
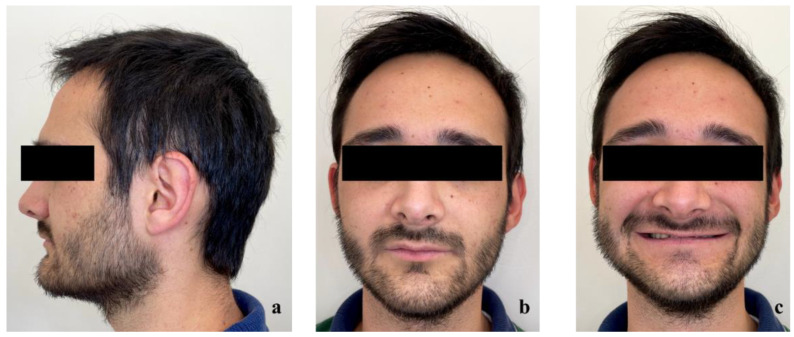
Extraoral examination: (**a**) profile view, (**b**) front view, (**c**) front view forced smile.

**Figure 2:**
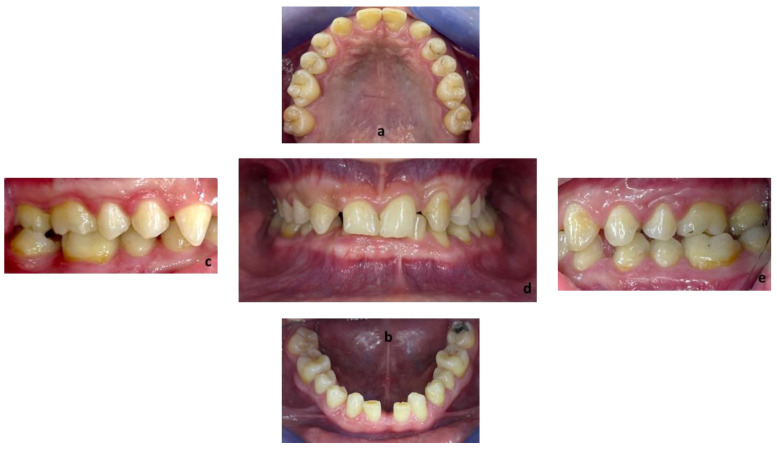
Endobuccal views: (**a**) maxillary arch, (**b**) mandibular arch, (**c**) right side occlusion, (**d**) front bite, (**e**) left side occlusion.

**Figure 3:**
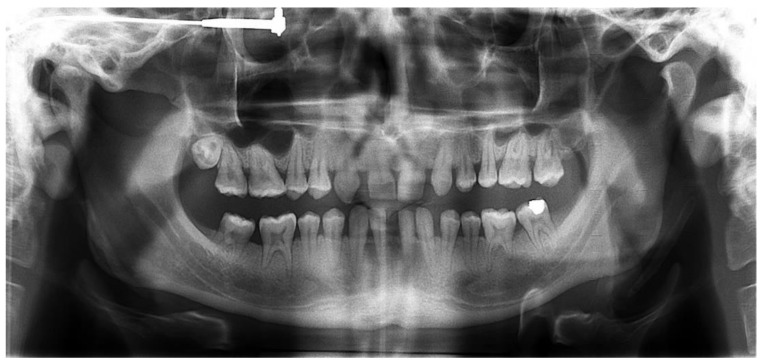
Orthopantomogram of the patient’s initial dental condition in February 2021.

First, alginate impressions were made to obtain diagnostic casts. These casts were mounted in an articulator in order to analyze the case. Face photos and intraoral views were taken and are presented in Figure 1 and Figure 2. The orthopantomogram shown in Figure 3 was realized.

Exobuccal clinical assessment showed a leftward convergence of the facial lines, an asymmetric smile, and the presence of oral corridors. The profile examination showed a lowering of the lower face, an open naso-labial angle, a concave subnasal profile with a reduced cervical-chin distance and a biretrochelia.

Endobuccal clinical assessment revealed dental agenesis of the upper left and right lateral incisors and lower left and right central incisors, generalized small teeth size, infiltrated maxillary grooves, and an amalgam restoration on the occlusal surface of the lower left second molar. Occlusal analysis showed complete overbite, a left unilateral articular inversion on the two upper left molars, an Angle Class I canine and molar on the right side and an Angle Class II canine and molar on the left side. In addition, the anterior overhang is reduced, so the patient is in class II division 2. 

The radiological examination revealed voluminous dental pulps, apical root resorptions on the lower molars and the absence of the upper left third molar and the two lower third molars.

According to the patient, his younger sister also suffers from agenesis (absence of seven teeth), but no genetic test has been conducted in the family. 

### 2.3. Therapeutic Objectives

To improve the quality of life and limit the psychological impact of his unattractive smile, it was necessary to restore aesthetics and function. The patient had previously undergone orthodontic treatment to close the spaces created by the agenesia. Therefore, implant treatment was not possible due to the lack of space and financial reasons. The patient did no longer wish to undergo orthodontic treatment, and the presence of significant apical resorption on the patient’s mandibular molars contraindicated further orthodontic treatment. In order to meet the patient’s aesthetic needs, it was decided to realize a full-mouth rehabilitation with a fixed prosthesis. In order to realize the fixed prosthesis, it was necessary to increase the vertical dimension of occlusion. The treatment consisted of a minimally invasive prosthetic procedure for the aesthetic rehabilitation of the complete arch. The objective was to preserve the enamel in order to optimize the adhesive bond of the luting agent to both the tooth surface and the ceramic restoration. Monolithic veneerlay (Emax, lithium disilicate) to the posterior teeth (upper and lower premolars and molars) and monolithic crown to the anterior teeth (upper and lower incisors) were proposed and accepted by the patient. The prosthetic challenge is to restore an aesthetic and functional smile while preserving as much as possible the structure of the teeth.

### 2.4. Treatment

**Figure 4:**
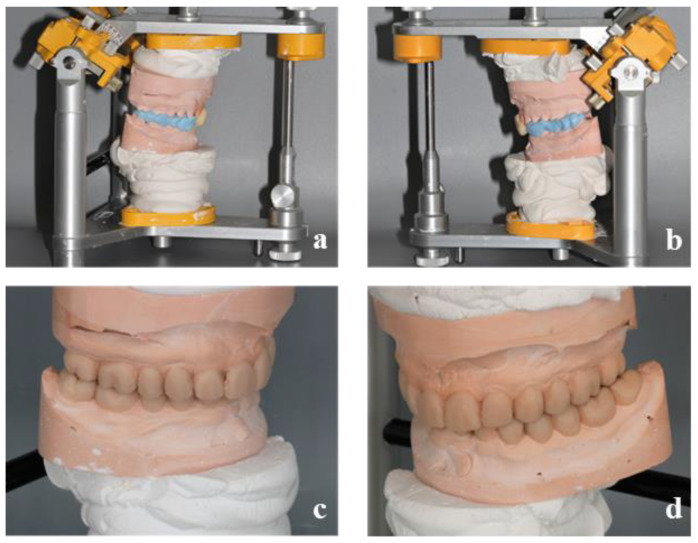
Placement in articulator of the models using the Lucia Jig and an occlusal bite (**a**,**b**) and wax-up of the models in the new vertical dimension of occlusion (**c**,**d**).

The centric relation was defined as a reference position to increase the vertical dimension of occlusion. It was recorded using an anterior deprogramming device (Lucia Jig technique), and then a silicone occlusion was placed posteriorly. The initial study casts were mounted at the new vertical dimension of occlusion (VDO) on a semi-adjustable articulator (SAM, semi-adjustable) using a facebow transfer for the maxillary and with the registration in centric relation for the mandible (Figure 4). The diagnostic wax-up was completed in accordance with the clinical findings. 

**Figure 5:**
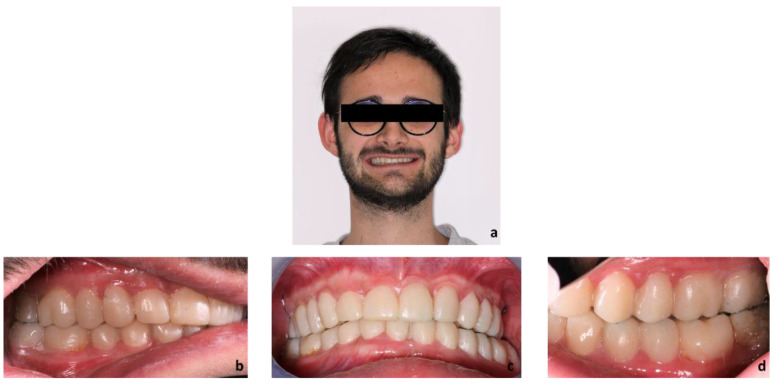
Exo (**a**) and endobuccal views with wax-up: (**b**) right side occlusion, (**c**) front bite, (**d**) left side occlusion.

These modifications were evaluated with a direct mock-up in the full-mouth prior to the initial preparation to evaluate aesthetics and function (Figure 5). The patient’s comfort, speech and appearance were assessed after 2 weeks.

Subsequently, an optical impression of the two arches with the composite resin mock-up was taken with intra-oral scanner Trios 3 (3Shape A/S, Copenhagen, Denmark). The Trios intra-oral scanner was operated on the confocal principle, with the video bitrate image capturing method based on the real-time rendering technique. The software 3Shape Dental Designer was used for modelling veenerlays and crowns.

**Figure 6:**
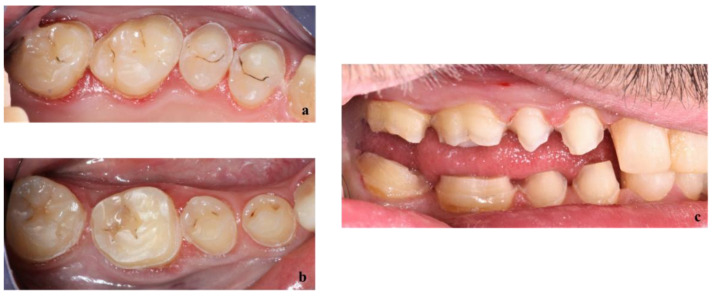
Final teeth preparations on the upper right side (**a**), on the lower right side (**b**), in occlusal view on the right side (**c**).

Once the volume of the final restoration was defined by the complete mock-up, the preparation of teeth for the definitive adhesive restoration could be performed with calibrated burs to achieve an occlusal reduction of 1.5 to 2 mm. A very light chamber preparation was made on posterior teeth in vestibular and proximal faces with a supragingival position, and a finish line on the cervical area of anterior teeth was positioned in the sulcus to optimize the aesthetic result (Figure 6). 

As a result, it was possible to maintain most of the enamel necessary for bonding. The bonding procedure followed a very strict operating protocol requiring the use of a rubber dam at each step. The inner surfaces of the crowns were etched with 9.5% hydrofluoric acid (IPS ceramic etching gel; Ivoclar Vivadent) for 20 s, rinsed, and air-dried. A silane-coupling agent (Monobond-Plus; Ivoclar-Vivadent) was then applied for 60 s. Subsequently, the surface was dried with free air. The veenerlays and crowns were individually luted, following the same sequence for each. The tooth surface was etched with 37% phosphoric acid for 15 s in dentin and 30 s in enamel, copiously rinsed, and carefully dried. Then, the adhesive was applied and light-cured with a Woodpecker LED-C curing light. The cementation was carried out using a light-curing composite cement (Variolink Veneer; Ivoclar Vivadent), which was applied to the inner surface of the indirect restoration. Each prosthetic component was carefully positioned and light-cured for 5 s. Excess cement was removed, and the restorations were light-cured for 40 s using a LED unit in vestibular and lingual/palatal surfaces. The remaining excess cement was removed with a scalpel blade, and the occlusion was checked. Finishing and polishing were performed with silicone rubber polishers.

The preparations and impressions were made in three separate sessions and by sector in order to always maintain the defined VDO. 

The management of dental treatment for the upper and lower right side is detailed as an example.

**Figure 7:**
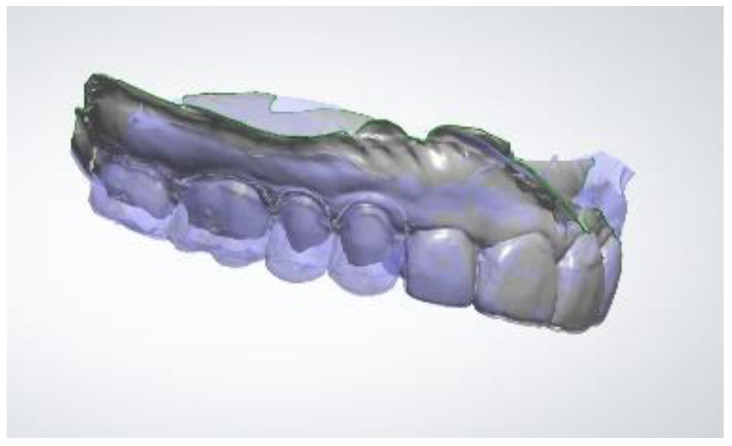
Virtual image of the recorded mock-up resulting from the optical impression.

Once the mock-up had been validated by the patient after 2 weeks, the first clinical session was to take an impression of the mock-up in the mouth using the Trios 3 intraoral scanner. The resulting image of the virtual working model is shown in Figure 7.

**Figure 8:**
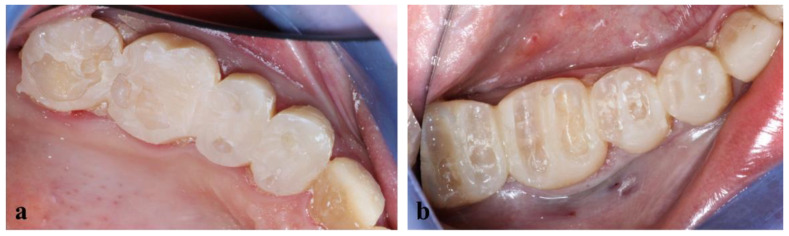
Teeth preparation through the mock-up of upper (**a**) and lower (**b**) right premolars and molars.

Teeth of the upper (a) and lower (b) right pre-molar and molar sectors were prepared through the mock-up as shown in Figure 8.

**Figure 9:**
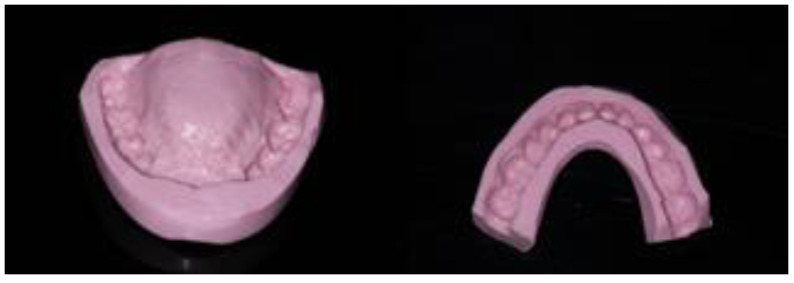
Silicone keys made for repositioning temporary resin after teeth preparations.

Once teeth were prepared, an optical impression of the preparations was made, and a temporary resin (Structur Premium, VOCO) was placed on the teeth with silicone keys (Figure 9) to protect them for a week, the time needed for the prosthetic laboratory to prepare the fixed prosthetic components.

**Figure 10:**
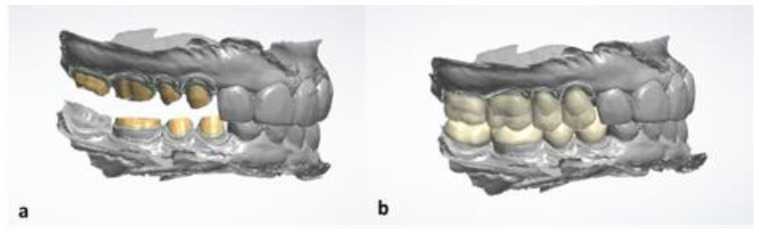
Teeth preparations on Dental Designer software (**a**), modeling of the prosthetic elements according to the mock-up (**b**).

A digital cast was obtained by intraoral scanning, and the quality of the preparations was reevaluated. Veenerlays and crowns were designed by using the Dental Designer software program. A laboratory modelled the prosthetic elements according to the mock-up, and then the design file was sent to the DWX—42 W machine from DG Shape. Figure 10 illustrates the teeth prepared on the virtual model (a) and the prosthetic elements according to the mock-up (b). 

The laboratory’s in-house dental technician applied make-up to the prosthetics components to enhance the aesthetic appearance and then applied the glaze layer, which was crystallized in an oven according to the manufacturer’s recommendations. 

**Figure 11:**
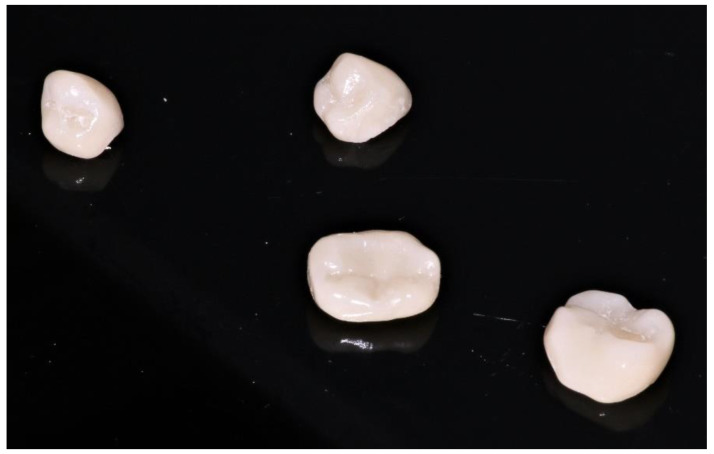
Fixed prosthetic elements traditionally glazed and polished.

During the second clinical session, the temporary resin was applied, the teeth were thoroughly cleaned, and the fixed prosthetic components (Figure 11) were tried on. 

**Figure 12:**
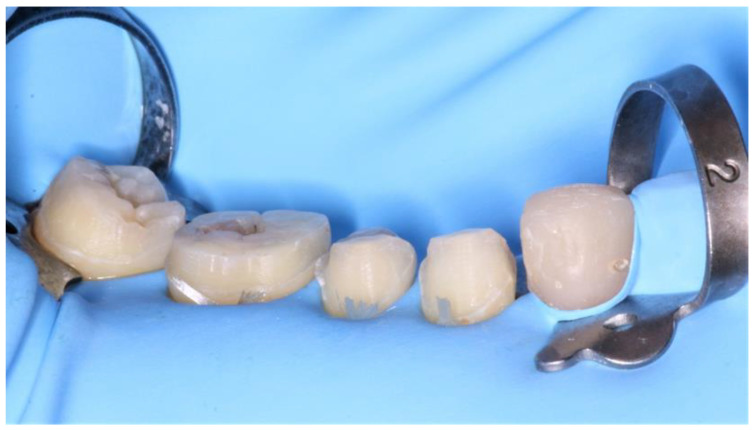
Installation of a rubber dam.

**Figure 13:**
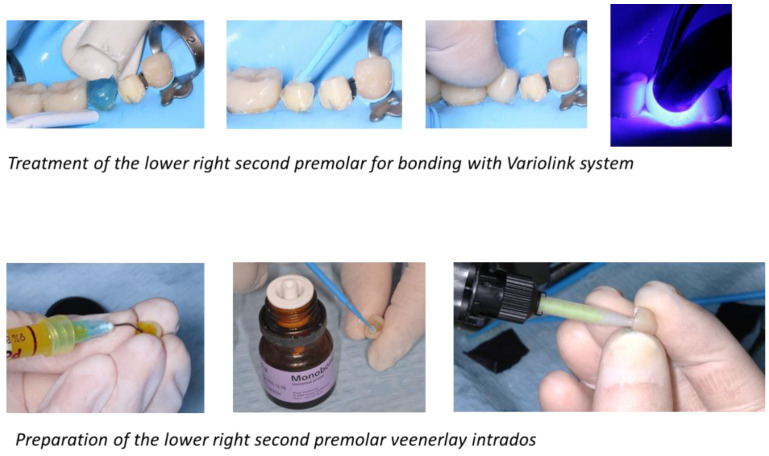
Bonding protocol for the lower right second premolar with Variolink system.

Once validated, a rubber dam was installed (Figure 12) to allow optimal bonding according to the protocol described above (Figure 13).

This second clinical session ended with the removal of the rubber dam and adjustments of the bonded prosthetic components (verification of marginal adaptation, proximal contact, occlusion, and aesthetics). Self-glazed monolithic veenerlays and crowns fabricated with the completely digital workflow provided efficient and satisfactory clinical performance.

**Figure 14:**
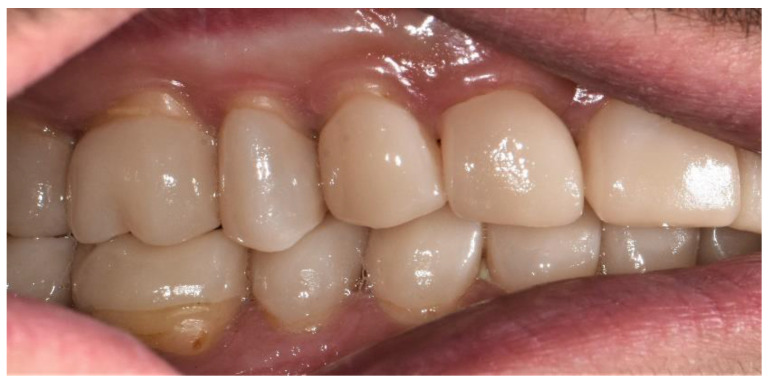
Final view of the right side.

The final view of the upper and lower right side is shown in Figure 14.

During treatment, the patient presented severe pain in the lower left second molar and lower right first molar that was not relieved by analgesics. After clinical examination, these two teeth were diagnosed with acute reversible pulpitis. A therapeutic pulpotomy treatment with Biodentine™ (Septodont) was performed through the overlay. The access cavity was filled with composite resin, and the occlusion was checked.

Treatment lasted a total of 7 weeks (one session per week) and was followed by two additional check-up appointments to respond to the patient’s grievances.

### 2.5. Results of Treatment

One month after the end of treatment, an increase in the GOHAI score, compared to the initial situation, was noted, with a score of 54/60. This final score, which corresponds to an adequate oral quality of life, was qualified according to the GOHAI scale. The Hue index (SD Hue) allowed the evaluation of masticatory function [10] and showed a significant decrease in heterogeneity before and after treatment. These results reflected an improvement in masticatory performance by the prosthetic treatment. Indeed, the SD Hue at five cycles varies from 0.64 before treatment to 0.23 after treatment. This decrease in SD Hue is also verified on chewing gums at 20 cycles and at 1 min. 

**Figure 15:**
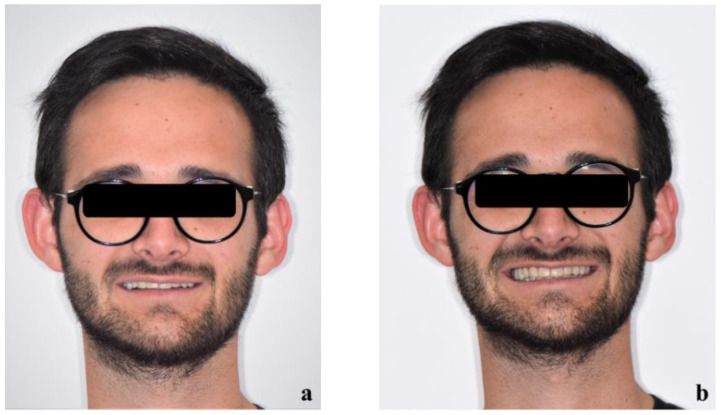
Extraoral examination at the end of the treatment: smile of the patient (**a**) and forced smile of the patient (**b**).

The patient’s new smile is shown in Figure 15.

**Figure 16:**
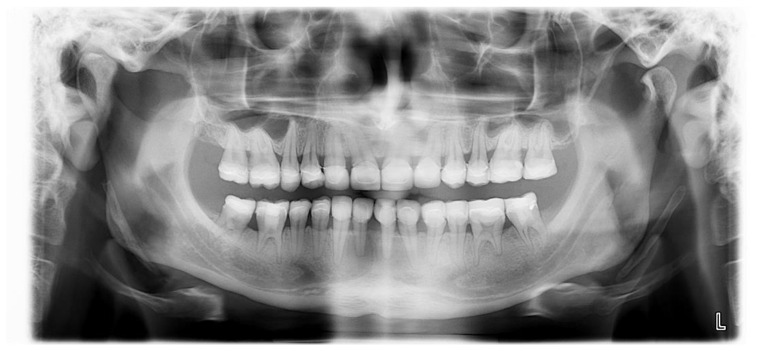
Orthopantomogram of the patient at 8 months after the end of the dental treatment.

The GOHAI was performed at 8 months after the end of treatment, and the score obtained was 59/60, indicating an excellent oral quality of life. An orthopantomogram of the patient at 8 months after the end of the dental treatment is presented in Figure 16.

## 3. Discussion

For this treatment, it was deliberately chosen not to restore the maxillary and mandibular canines at the anterior level. Indeed, the lack of space led us to prioritize the smile by reconstituting the eight incisors in order to obtain the best aesthetic result considering the clinical situation of the subject. Additionally, for aesthetic reasons, we chose to perform vestibular preparations on the posterior teeth. Following this treatment, the lower left second molar and lower right first molar underwent a therapeutic pulpotomy [11,12] that could have been avoided by making an overlay design without a buccal veneer on the posterior teeth [13]. Indeed, an in vitro study conducted by Monaco et al. showed that the fabrication of dental table-top preparations requires a very small reduction in tissue [14]. In our case, the vertical dimension was increased, and a very light occlusal preparation would have been necessary to carry out the restoration of the posterior teeth by using table-top preparation to avoid performing the therapeutic pulpotomies. However, our patient’s high aesthetic demands led us to cover the vestibular surface of the teeth. 

There has been a growing interest in glass-ceramic systems with good aesthetics, high fracture resistance and bonding durability, and simplified fabrication techniques using CAD/CAM. The choice of material for full-mouth restoration in this case was lithium disilicate glass-ceramic (e-MAX, Ivoclar Vivadent). Lithium disilicate glass-ceramic has better mechanical and excellent optical properties than conventional dental porcelain. It has become popular in a monolithic configuration [15,16]. Single crowns made of either lithium disilicate ceramic have been reported to have good clinical longevity similar to metal-ceramic single crowns. Advantages of monolithic single crowns include their ability to resist applied masticatory stresses by absorbing and dissipating them through the entire restoration, as stress concentration is related to flaws, porosities of the surfaces, and integral inhomogeneities [17,18]. In this clinical case, the use of adhesive dentistry made it possible to dispense with retentive peripheral preparations. Retention is ensured by chemical and micromechanical adhesion. Thus, significant tissue saving could be achieved thanks to minimal preparation [19].

This case report shows the interest of a CAD/CAM procedure during oral rehabilitation in a patient with agenesis by raising the vertical dimension and being minimally invasive. 

The interest of the CAD/CAM with the same procedure has already been shown in extensive erosive lesions [20,21] and traumatology [22]. For this patient, an improvement in masticatory performance is clearly visible. After treatment, the SD Hue at five cycles is largely reduced, reflecting greater homogeneity of the chewed gum. It is interesting to note an improvement in the patient’s functional aspect after treatment, even though his initial request was only aesthetic. Furthermore, the treatment resulted in a significant improvement in the patient’s oral quality of life, directly after the end of treatment and after 8 months. An improvement in the patient’s aesthetic consideration can be noted directly after the placement of the prostheses. Similar results have also been shown in geriatric and pediatric dentistry but poorly described in the treatment of special needs patients: CAD/CAM procedure is rarely described in special care dentistry, although its use is promising from both an aesthetic and functional point of view [23,24,25]. 

The long-term prognosis is favorable and conditioned upon good oral health hygiene. According to Abduo et al. and Aldegheishem et al., this kind of restoration has a high survival rate at 5 years [26,27].

## 4. Conclusions

This clinical case is an example of functional and aesthetic rehabilitation in a patient with generalized small teeth size and multiple agenesia. The use of CAD/CAM systems has allowed time saving, tissue saving, better communication and aesthetic management with the patient during a complex prosthetic treatment.
